# 8-Methyl-2,3,4,9-tetra­hydro-1*H*-carbazol-1-one

**DOI:** 10.1107/S1600536810031545

**Published:** 2010-08-11

**Authors:** R. Archana, E. Yamuna, K. J. Rajendra Prasad, A. Thiruvalluvar, R. J. Butcher

**Affiliations:** a PG Research Department of Physics, Rajah Serfoji Government College (Autonomous), Thanjavur 613 005, Tamilnadu, India; b Department of Chemistry, Bharathiar University, Coimbatore 641 046, Tamilnadu, India; c Department of Chemistry, Howard University, 525 College Street NW, Washington, DC 20059, USA

## Abstract

In the title compound, C_13_H_13_NO, the dihedral angle between the benzene ring and the fused pyrrole ring is 0.96 (7)°. The cyclohexenone ring adopts an envelope conformation. Inter­molecular N—H⋯O hydrogen bonds form *R* ^2^_2_(10) ring motifs in the crystal structure. Weak C—H⋯π inter­actions involving the benzene ring also occur.

## Related literature

For tetra­hydro­carbazolones, see: Bringmann *et al.* (1995 ▶); Chakravarty *et al.* (2001 ▶); Knölker & Reddy (2002 ▶); Lin & Zhang (2000 ▶); Matsuo & Ishida (1994 ▶); Miki & Hachiken (1993 ▶); Scott *et al.* (2006 ▶). For biologically active carbazoles, see: Jean *et al.* (2004 ▶); Knölker & Reddy (2008 ▶). For the preparation of 1-oxo compounds *via* their corresponding hydrazones, see: Rajendra Prasad & Vijayalakshmi (1994 ▶). For crystal structures of substituted carbazole derivatives, see: Thomas Gunaseelan *et al.* (2009 ▶); Sridharan *et al.* (2008 ▶); Thiruvalluvar *et al.* (2007 ▶). For ring conformations, see: Cremer & Pople (1975 ▶). For hydrogen-bond motifs, see: Bernstein *et al.* (1995 ▶).
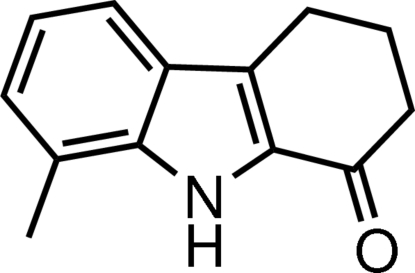


## Experimental

### 

#### Crystal data

C_13_H_13_NO
*M* *_r_* = 199.24
Monoclinic, 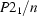

*a* = 10.5245 (2) Å
*b* = 7.1564 (1) Å
*c* = 13.5870 (3) Å
β = 93.960 (2)°
*V* = 1020.90 (3) Å^3^
*Z* = 4
Cu *K*α radiation
μ = 0.65 mm^−1^
*T* = 110 K
0.51 × 0.42 × 0.34 mm

#### Data collection

Oxford Diffraction Xcalibur Ruby Gemini diffractometer
Absorption correction: multi-scan (*CrysAlis PRO*; Oxford Diffraction, 2009 ▶) *T*_min_ = 0.751, *T*_max_ = 1.000
3655 measured reflections
2005 independent reflections
1882 reflections with *I* > 2σ(*I*)
*R*_int_ = 0.016

#### Refinement

*R*[*F* ^2^ > 2σ(*F* ^2^)] = 0.044
*wR*(*F* ^2^) = 0.120
*S* = 1.05
2005 reflections
141 parameters
H atoms treated by a mixture of independent and constrained refinement
Δρ_max_ = 0.33 e Å^−3^
Δρ_min_ = −0.30 e Å^−3^

### 

Data collection: *CrysAlis PRO* (Oxford Diffraction, 2009 ▶); cell refinement: *CrysAlis PRO*; data reduction: *CrysAlis PRO*; program(s) used to solve structure: *SHELXS97* (Sheldrick, 2008 ▶); program(s) used to refine structure: *SHELXL97* (Sheldrick, 2008 ▶); molecular graphics: *ORTEP-3* (Farrugia, 1997 ▶); software used to prepare material for publication: *PLATON* (Spek, 2009 ▶).

## Supplementary Material

Crystal structure: contains datablocks global, I. DOI: 10.1107/S1600536810031545/jj2046sup1.cif

Structure factors: contains datablocks I. DOI: 10.1107/S1600536810031545/jj2046Isup2.hkl

Additional supplementary materials:  crystallographic information; 3D view; checkCIF report

## Figures and Tables

**Table 1:** Hydrogen-bond geometry (Å, °) *Cg*3 is the centroid of the C4*B*,C5–C8,C8*A* ring.

*D*—H⋯*A*	*D*—H	H⋯*A*	*D*⋯*A*	*D*—H⋯*A*
N9—H9⋯O1^i^	0.87 (2)	2.01 (2)	2.8603 (15)	165.9 (18)
C2—H2*B*⋯*Cg*3^ii^	0.99	2.64	3.5429 (13)	152
C5—H5⋯*Cg*3^iii^	0.95	2.86	3.6414 (14)	140

Symmetry codes: (i) 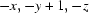; (ii) 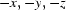; (iii) 
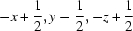
.

**Fig. 1.**
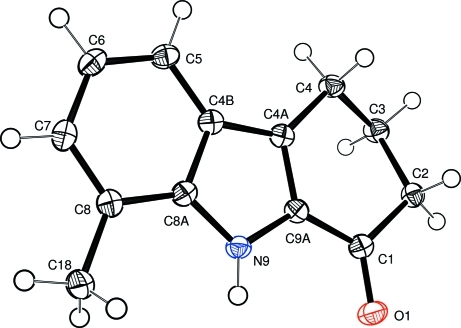
The molecular structure of the title compound, showing the atom-numbering scheme and displacement ellipsoids drawn at the 50% probability level. H atoms are shown as small spheres of arbitrary radius.

**Fig. 2.**
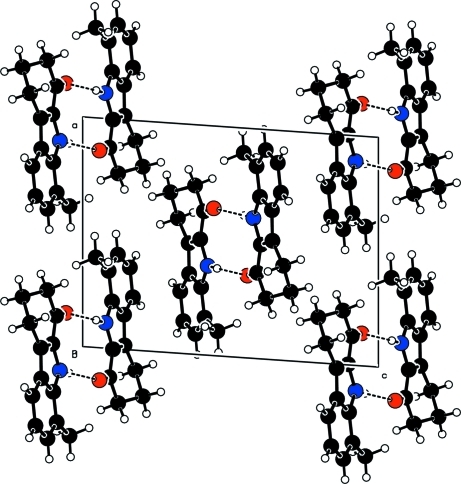
The crystal structure of (I), viewed down the b axis, showing the formation of a R22(10) ring motif.

## supplementary crystallographic information

### Comment

Tetrahydrocarbazolones have been used extensively as advanced intermediates in synthetic efforts toward a number of naturally occurring carbazole alkaloids (Bringmann *et al.*, (1995); Chakravarty *et al.*, (2001); Knölker & Reddy (2002); Lin & Zhang (2000); Matsuo & Ishida (1994); Miki & Hachiken (1993); Scott *et al.*, (2006)). Jean *et al.* (2004) and Knölker & Reddy (2008) have reported biologically active carbazoles. The preparation of 1-oxo compounds *via* their corresponding hydrazones have been reported (Rajendra Prasad & Vijayalakshmi (1994)).

Thomas Gunaseelan *et al.* (2009), Sridharan *et al.* (2008) and Thiruvalluvar *et al.* (2007) have reported the crystal structures of substituted carbazole derivatives, in which the carbazole units are not planar. In the title molecule (Scheme I, Fig. 1), C_13_H_13_NO, the carbazole unit is not planar. The dihedral angle between the benzene ring and the fused pyrrole ring is 0.96 (7)°. The r.m.s. deviation of a mean plane fitted through all non hydrogen atoms excluding C3 of the carbazole unit is 0.0180 Å; C3 deviates from this plane by 0.620 (2) Å. The cyclohexene ring adopts an envelope conformation. The puckering parameters (Cremer & Pople, 1975) are q2=0.3623 (14) Å, q3=-0.2730 (14) Å, Q=0.4536 (14) Å, θ=127.00 (18)° and φ=292.7 (2)°. Intermolecular N9—H9···O1 hydrogen bonds form a *R*^2^_2_(10)(Bernstein *et al.*, 1995) ring motif in the crystal structure (Table 1, Fig. 2). Weak C2—H2B···π and C5—H5···π interactions involving the benzene (C4B,C5—C8,C8A) ring are also found in the structure(Table 1).

### Experimental

A solution of 2-(2-*o*-tolylhydrazono)-cyclohexanone (0.216 g, 0.001 mol) in a mixture of acetic acid (20 ml) and hydrochloric acid (5 ml) was refluxed on an oil bath pre-heated to 398 K for 2 h. The contents were then cooled and poured onto cold water with stirring. The brown solid which was separated by passing through a column of silica gel and eluted with (98:2, *v*/*v*) petroleum ether: ethyl acetate mixture to yield the title compound (0.126 g, 63%). This was recrystallized from ethanol.

### Refinement

The H atom bonded to N9 was located in a difference Fourier map and refined freely. Other H atoms were positioned geometrically and allowed to ride on their parent atoms, with C—H = 0.95–0.99 Å and *U*_iso_(H) = 1.2–1.5*U*_eq_(parent atom).

### Crystal data

**Table:**

C_13_H_13_NO	*F*(000) = 424
*M**_r_* = 199.24	*D*_x_ = 1.296 Mg m^−^^3^
Monoclinic, *P*2_1_/*n*	Melting point: 443 K
Hall symbol: -P 2yn	Cu *K*α radiation, λ = 1.54184 Å
*a* = 10.5245 (2) Å	Cell parameters from 3169 reflections
*b* = 7.1564 (1) Å	θ = 5.5–74.0°
*c* = 13.5870 (3) Å	µ = 0.65 mm^−^^1^
β = 93.960 (2)°	*T* = 110 K
*V* = 1020.90 (3) Å^3^	Prism, colourless
*Z* = 4	0.51 × 0.42 × 0.34 mm

### Data collection

**Table:**

Oxford Diffraction Xcalibur Ruby Gemini diffractometer	2005 independent reflections
Radiation source: Enhance (Cu) X-ray Source	1882 reflections with *I* > 2σ(*I*)
graphite	*R*_int_ = 0.016
Detector resolution: 10.5081 pixels mm^-1^	θ_max_ = 74.1°, θ_min_ = 5.5°
ω scans	*h* = −13→12
Absorption correction: multi-scan (*CrysAlis PRO*; Oxford Diffraction, 2009)	*k* = −8→8
*T*_min_ = 0.751, *T*_max_ = 1.000	*l* = −16→12
3655 measured reflections	

### Refinement

**Table:**

Refinement on *F*^2^	Primary atom site location: structure-invariant direct methods
Least-squares matrix: full	Secondary atom site location: difference Fourier map
*R*[*F*^2^ > 2σ(*F*^2^)] = 0.044	Hydrogen site location: inferred from neighbouring sites
*wR*(*F*^2^) = 0.120	H atoms treated by a mixture of independent and constrained refinement
*S* = 1.05	*w* = 1/[σ^2^(*F*_o_^2^) + (0.0737*P*)^2^ + 0.4222*P*] where *P* = (*F*_o_^2^ + 2*F*_c_^2^)/3
2005 reflections	(Δ/σ)_max_ = 0.001
141 parameters	Δρ_max_ = 0.33 e Å^−^^3^
0 restraints	Δρ_min_ = −0.30 e Å^−^^3^

### Special details

**Table:**

Geometry. Bond distances, angles *etc*. have been calculated using the rounded fractional coordinates. All su's are estimated from the variances of the (full) variance-covariance matrix. The cell e.s.d.'s are taken into account in the estimation of distances, angles and torsion angles
Refinement. Refinement of *F*^2^ against ALL reflections. The weighted *R*-factor *wR* and goodness of fit *S* are based on *F*^2^, conventional *R*-factors *R* are based on *F*, with *F* set to zero for negative *F*^2^. The threshold expression of *F*^2^ > 2σ(*F*^2^) is used only for calculating *R*-factors(gt) *etc*. and is not relevant to the choice of reflections for refinement. *R*-factors based on *F*^2^ are statistically about twice as large as those based on *F*, and *R*- factors based on ALL data will be even larger.

### Fractional atomic coordinates and isotropic or equivalent isotropic displacement parameters (Å^2^)

**Table:**

	*x*	*y*	*z*	*U*_iso_*/*U*_eq_	
O1	−0.14139 (9)	0.43448 (14)	0.05682 (7)	0.0252 (3)	
N9	0.11062 (10)	0.26331 (16)	0.07724 (8)	0.0180 (3)	
C1	−0.12572 (12)	0.27613 (18)	0.09016 (9)	0.0180 (3)	
C2	−0.23534 (12)	0.15732 (18)	0.11989 (9)	0.0188 (3)	
C3	−0.19848 (12)	0.01890 (19)	0.20323 (10)	0.0211 (4)	
C4	−0.08735 (12)	−0.10760 (18)	0.17805 (9)	0.0202 (3)	
C4A	0.01788 (12)	0.00943 (17)	0.14160 (9)	0.0171 (3)	
C4B	0.15114 (12)	−0.02537 (18)	0.13993 (9)	0.0177 (3)	
C5	0.22998 (13)	−0.17717 (18)	0.16994 (9)	0.0207 (4)	
C6	0.35880 (13)	−0.16125 (19)	0.15961 (10)	0.0229 (4)	
C7	0.41100 (12)	0.00095 (19)	0.11902 (10)	0.0215 (4)	
C8	0.33738 (12)	0.15274 (18)	0.08785 (9)	0.0194 (4)	
C8A	0.20563 (12)	0.13615 (17)	0.09939 (9)	0.0174 (3)	
C9A	−0.00264 (12)	0.18558 (18)	0.10250 (9)	0.0171 (3)	
C18	0.39245 (13)	0.3254 (2)	0.04456 (11)	0.0274 (4)	
H2A	−0.30348	0.24034	0.14124	0.0226*	
H2B	−0.27040	0.08665	0.06153	0.0226*	
H3A	−0.17410	0.08918	0.26440	0.0254*	
H3B	−0.27315	−0.05960	0.21569	0.0254*	
H4A	−0.11694	−0.19866	0.12660	0.0242*	
H4B	−0.05557	−0.17803	0.23745	0.0242*	
H5	0.19525	−0.28723	0.19651	0.0248*	
H6	0.41364	−0.26137	0.18020	0.0275*	
H7	0.50030	0.00602	0.11288	0.0258*	
H9	0.1191 (16)	0.367 (3)	0.0455 (13)	0.028 (4)*	
H18A	0.48445	0.30932	0.04072	0.0411*	
H18B	0.35236	0.34649	−0.02177	0.0411*	
H18C	0.37648	0.43317	0.08648	0.0411*	

### Atomic displacement parameters (Å^2^)

**Table:**

	*U*^11^	*U*^22^	*U*^33^	*U*^12^	*U*^13^	*U*^23^
O1	0.0232 (5)	0.0218 (5)	0.0312 (5)	0.0037 (4)	0.0057 (4)	0.0081 (4)
N9	0.0179 (5)	0.0162 (5)	0.0203 (5)	0.0005 (4)	0.0034 (4)	0.0027 (4)
C1	0.0204 (6)	0.0198 (6)	0.0140 (6)	0.0004 (5)	0.0021 (4)	−0.0001 (5)
C2	0.0171 (6)	0.0201 (6)	0.0195 (6)	0.0003 (5)	0.0026 (5)	−0.0010 (5)
C3	0.0209 (6)	0.0217 (7)	0.0214 (6)	−0.0013 (5)	0.0059 (5)	0.0027 (5)
C4	0.0218 (6)	0.0181 (6)	0.0210 (6)	−0.0010 (5)	0.0042 (5)	0.0034 (5)
C4A	0.0201 (6)	0.0178 (6)	0.0136 (6)	−0.0005 (5)	0.0019 (4)	−0.0002 (4)
C4B	0.0211 (6)	0.0186 (6)	0.0133 (6)	0.0003 (5)	0.0016 (4)	−0.0008 (4)
C5	0.0252 (7)	0.0189 (6)	0.0179 (6)	0.0016 (5)	0.0013 (5)	0.0030 (5)
C6	0.0239 (7)	0.0224 (7)	0.0220 (6)	0.0060 (5)	−0.0017 (5)	0.0018 (5)
C7	0.0173 (6)	0.0260 (7)	0.0209 (6)	0.0022 (5)	−0.0003 (5)	−0.0019 (5)
C8	0.0193 (6)	0.0209 (7)	0.0181 (6)	−0.0005 (5)	0.0016 (5)	−0.0017 (5)
C8A	0.0196 (6)	0.0181 (6)	0.0145 (6)	0.0014 (5)	0.0006 (4)	−0.0008 (4)
C9A	0.0179 (6)	0.0183 (6)	0.0154 (6)	−0.0010 (5)	0.0031 (4)	0.0001 (4)
C18	0.0191 (7)	0.0248 (7)	0.0385 (8)	−0.0009 (5)	0.0045 (5)	0.0046 (6)

### Geometric parameters (Å, °)

**Table:**

O1—C1	1.2273 (16)	C7—C8	1.3836 (18)
N9—C8A	1.3700 (17)	C8—C8A	1.4112 (18)
N9—C9A	1.3801 (17)	C8—C18	1.5019 (19)
N9—H9	0.87 (2)	C2—H2A	0.9900
C1—C9A	1.4478 (18)	C2—H2B	0.9900
C1—C2	1.5105 (18)	C3—H3A	0.9900
C2—C3	1.5340 (18)	C3—H3B	0.9900
C3—C4	1.5362 (18)	C4—H4A	0.9900
C4—C4A	1.4997 (18)	C4—H4B	0.9900
C4A—C4B	1.4262 (18)	C5—H5	0.9500
C4A—C9A	1.3792 (18)	C6—H6	0.9500
C4B—C8A	1.4185 (18)	C7—H7	0.9500
C4B—C5	1.4100 (18)	C18—H18A	0.9800
C5—C6	1.3773 (19)	C18—H18B	0.9800
C6—C7	1.4125 (19)	C18—H18C	0.9800
			
O1···N9	2.9173 (14)	H2A···H18A^vii^	2.5800
O1···C18^i^	3.3659 (17)	H2A···C3^vi^	2.9000
O1···N9^i^	2.8603 (15)	H2A···C4^vi^	2.9900
O1···H9	2.798 (17)	H2A···H3B^vi^	2.5000
O1···H4A^ii^	2.8000	H2A···H4B^vi^	2.3700
O1···H9^i^	2.01 (2)	H2B···C7^iii^	2.8500
O1···H18B^i^	2.7300	H2B···C8^iii^	2.7100
N9···O1	2.9173 (14)	H2B···C8A^iii^	2.8300
N9···O1^i^	2.8603 (15)	H3A···C9A	3.0200
N9···H4A^iii^	2.8100	H3A···H3B^vi^	2.5900
C1···C4B^iii^	3.5986 (18)	H3B···C2^viii^	3.0100
C4A···C9A^iii^	3.5916 (17)	H3B···H2A^viii^	2.5000
C4B···C1^iii^	3.5986 (18)	H3B···H3A^viii^	2.5900
C5···C8A^iv^	3.4312 (17)	H4A···O1^ix^	2.8000
C8A···C5^v^	3.4312 (17)	H4A···N9^iii^	2.8100
C9A···C4A^iii^	3.5916 (17)	H4B···H2A^viii^	2.3700
C18···O1^i^	3.3659 (17)	H5···C8^iv^	3.0000
C2···H3B^vi^	3.0100	H5···C8A^iv^	2.9500
C2···H7^vii^	2.9800	H6···H18C^ix^	2.5500
C3···H2A^viii^	2.9000	H6···C4A^iv^	2.9700
C4···H2A^viii^	2.9900	H6···C9A^iv^	3.0600
C4A···H6^v^	2.9700	H7···C2^x^	2.9800
C6···H18C^ix^	3.0800	H7···H18A	2.3800
C7···H2B^iii^	2.8500	H9···O1	2.798 (17)
C8···H2B^iii^	2.7100	H9···C18	2.893 (17)
C8···H5^v^	3.0000	H9···O1^i^	2.01 (2)
C8A···H5^v^	2.9500	H18A···H2A^x^	2.5800
C8A···H2B^iii^	2.8300	H18A···H7	2.3800
C9A···H3A	3.0200	H18B···O1^i^	2.7300
C9A···H6^v^	3.0600	H18C···C6^ii^	3.0800
C18···H9	2.893 (17)	H18C···H6^ii^	2.5500
			
C8A—N9—C9A	107.91 (11)	C1—C2—H2A	109.00
C9A—N9—H9	126.1 (11)	C1—C2—H2B	109.00
C8A—N9—H9	125.4 (11)	C3—C2—H2A	109.00
O1—C1—C9A	123.53 (12)	C3—C2—H2B	109.00
O1—C1—C2	122.20 (11)	H2A—C2—H2B	108.00
C2—C1—C9A	114.26 (11)	C2—C3—H3A	109.00
C1—C2—C3	113.70 (10)	C2—C3—H3B	109.00
C2—C3—C4	111.95 (11)	C4—C3—H3A	109.00
C3—C4—C4A	109.60 (10)	C4—C3—H3B	109.00
C4B—C4A—C9A	106.35 (11)	H3A—C3—H3B	108.00
C4—C4A—C4B	131.09 (11)	C3—C4—H4A	110.00
C4—C4A—C9A	122.55 (11)	C3—C4—H4B	110.00
C5—C4B—C8A	119.64 (12)	C4A—C4—H4A	110.00
C4A—C4B—C8A	106.76 (11)	C4A—C4—H4B	110.00
C4A—C4B—C5	133.60 (12)	H4A—C4—H4B	108.00
C4B—C5—C6	118.07 (12)	C4B—C5—H5	121.00
C5—C6—C7	121.33 (12)	C6—C5—H5	121.00
C6—C7—C8	122.68 (12)	C5—C6—H6	119.00
C7—C8—C18	122.87 (12)	C7—C6—H6	119.00
C7—C8—C8A	115.70 (11)	C6—C7—H7	119.00
C8A—C8—C18	121.43 (11)	C8—C7—H7	119.00
N9—C8A—C8	128.87 (11)	C8—C18—H18A	109.00
C4B—C8A—C8	122.58 (11)	C8—C18—H18B	109.00
N9—C8A—C4B	108.55 (11)	C8—C18—H18C	109.00
C1—C9A—C4A	124.67 (12)	H18A—C18—H18B	109.00
N9—C9A—C1	124.89 (11)	H18A—C18—H18C	109.00
N9—C9A—C4A	110.44 (11)	H18B—C18—H18C	109.00
			
C9A—N9—C8A—C4B	−0.22 (14)	C4—C4A—C9A—N9	178.50 (11)
C9A—N9—C8A—C8	−179.22 (12)	C4—C4A—C9A—C1	−1.27 (19)
C8A—N9—C9A—C1	−179.79 (12)	C4B—C4A—C9A—N9	−0.48 (14)
C8A—N9—C9A—C4A	0.45 (14)	C4B—C4A—C9A—C1	179.75 (12)
O1—C1—C2—C3	−150.62 (12)	C4A—C4B—C5—C6	−178.27 (13)
C9A—C1—C2—C3	30.63 (15)	C8A—C4B—C5—C6	0.78 (18)
O1—C1—C9A—N9	−1.1 (2)	C4A—C4B—C8A—N9	−0.07 (14)
O1—C1—C9A—C4A	178.62 (12)	C4A—C4B—C8A—C8	179.00 (11)
C2—C1—C9A—N9	177.62 (11)	C5—C4B—C8A—N9	−179.35 (11)
C2—C1—C9A—C4A	−2.64 (18)	C5—C4B—C8A—C8	−0.28 (19)
C1—C2—C3—C4	−55.14 (14)	C4B—C5—C6—C7	−0.81 (19)
C2—C3—C4—C4A	48.86 (14)	C5—C6—C7—C8	0.3 (2)
C3—C4—C4A—C4B	156.41 (13)	C6—C7—C8—C8A	0.20 (19)
C3—C4—C4A—C9A	−22.29 (16)	C6—C7—C8—C18	−179.93 (14)
C4—C4A—C4B—C5	0.6 (2)	C7—C8—C8A—N9	178.66 (12)
C4—C4A—C4B—C8A	−178.52 (12)	C7—C8—C8A—C4B	−0.21 (18)
C9A—C4A—C4B—C5	179.47 (14)	C18—C8—C8A—N9	−1.2 (2)
C9A—C4A—C4B—C8A	0.33 (14)	C18—C8—C8A—C4B	179.92 (12)

Symmetry codes: (i) −*x*, −*y*+1, −*z*; (ii) *x*, *y*+1, *z*; (iii) −*x*, −*y*, −*z*; (iv) −*x*+1/2, *y*−1/2, −*z*+1/2; (v) −*x*+1/2, *y*+1/2, −*z*+1/2; (vi) −*x*−1/2, *y*+1/2, −*z*+1/2; (vii) *x*−1, *y*, *z*; (viii) −*x*−1/2, *y*−1/2, −*z*+1/2; (ix) *x*, *y*−1, *z*; (x) *x*+1, *y*, *z*.

### Hydrogen-bond geometry (Å, °)

**Table:**

Cg3 is the centroid of the C4B,C5–C8,C8A ring.

**Table:**

*D*—H···*A*	*D*—H	H···*A*	*D*···*A*	*D*—H···*A*
N9—H9···O1^i^	0.87 (2)	2.01 (2)	2.8603 (15)	165.9 (18)
C2—H2B···Cg3^iii^	0.99	2.64	3.5429 (13)	152
C5—H5···Cg3^iv^	0.95	2.86	3.6414 (14)	140

Symmetry codes: (i) −*x*, −*y*+1, −*z*; (iii) −*x*, −*y*, −*z*; (iv) −*x*+1/2, *y*−1/2, −*z*+1/2.
